# A novel mosaic variant on *SMC1A* reported in buccal mucosa cells, albeit not in blood, of a patient with Cornelia de Lange–like presentation

**DOI:** 10.1101/mcs.a005322

**Published:** 2020-06

**Authors:** Aixa Gonzalez Garcia, Julia Malone, Hong Li

**Affiliations:** 1 Department of Human Genetics, Emory University School of Medicine, Atlanta, Georgia 30322, USA;; 2 Department of Pediatrics, Emory University School of Medicine, Atlanta, Georgia 30322, USA

**Keywords:** delayed gross motor development, downturned corners of mouth, growth hormone deficiency, long upper eyelashes, lumbosacral hirsutism, microcephaly, moderate expressive language delay, synophrys

## Abstract

Mosaicism in Cornelia de Lange syndrome (CdLS) has been reported in clinically diagnosed CdLS patients with negative molecular testing using blood as the specimen, particularly in the *NIPBL* gene. Here we report a novel mosaic variant in *SMC1A* identified in the buccal swab DNA of a patient with a mild CdLS phenotype. Our patient presented with global developmental delay, dysmorphic features, microcephaly, and short stature, with no limb defect. Face2Gene, a digital tool that analyzes facial morphology, demonstrated a 97% match between our patient and the CdLS gestalt. An initial next-generation sequencing (NGS)-based CdLS panel test, including *NIPBL*, *HDAC8*, *RAD21*, *SMC1A*, and *SMC3*, completed using DNA isolated from leukocytes, was negative, and subsequent trio exome sequencing was nondiagnostic. The exome identified biallelic variants of uncertain significance in a candidate gene, *NSMCE2*. In the pursuit of a molecular diagnosis, a second NGS-based CdLS panel test was ordered on a buccal swab specimen and a novel variant, c.793_795delGAG (p.Glu265del) in *SMC1A*, was detected at 60% mosaicism. Retrospective analysis of the former panel and exome data revealed the *SMC1A* variant at 4% and 2%, respectively, both far below standard reporting thresholds. Given that mosaicism has been frequently reported in CdLS, we suggest selecting a different tissue for testing in clinically suspected CdLS cases, even after negative molecular results via blood specimen.

## CASE PRESENTATION

Here we present a 6-yr-old male with global developmental delays, microcephaly, short stature improved by growth hormone, small penis for age, plus hyperlipidemia, and dysmorphic features reminiscent of Cornelia de Lange syndrome (CdLS).

**Figure 1.**
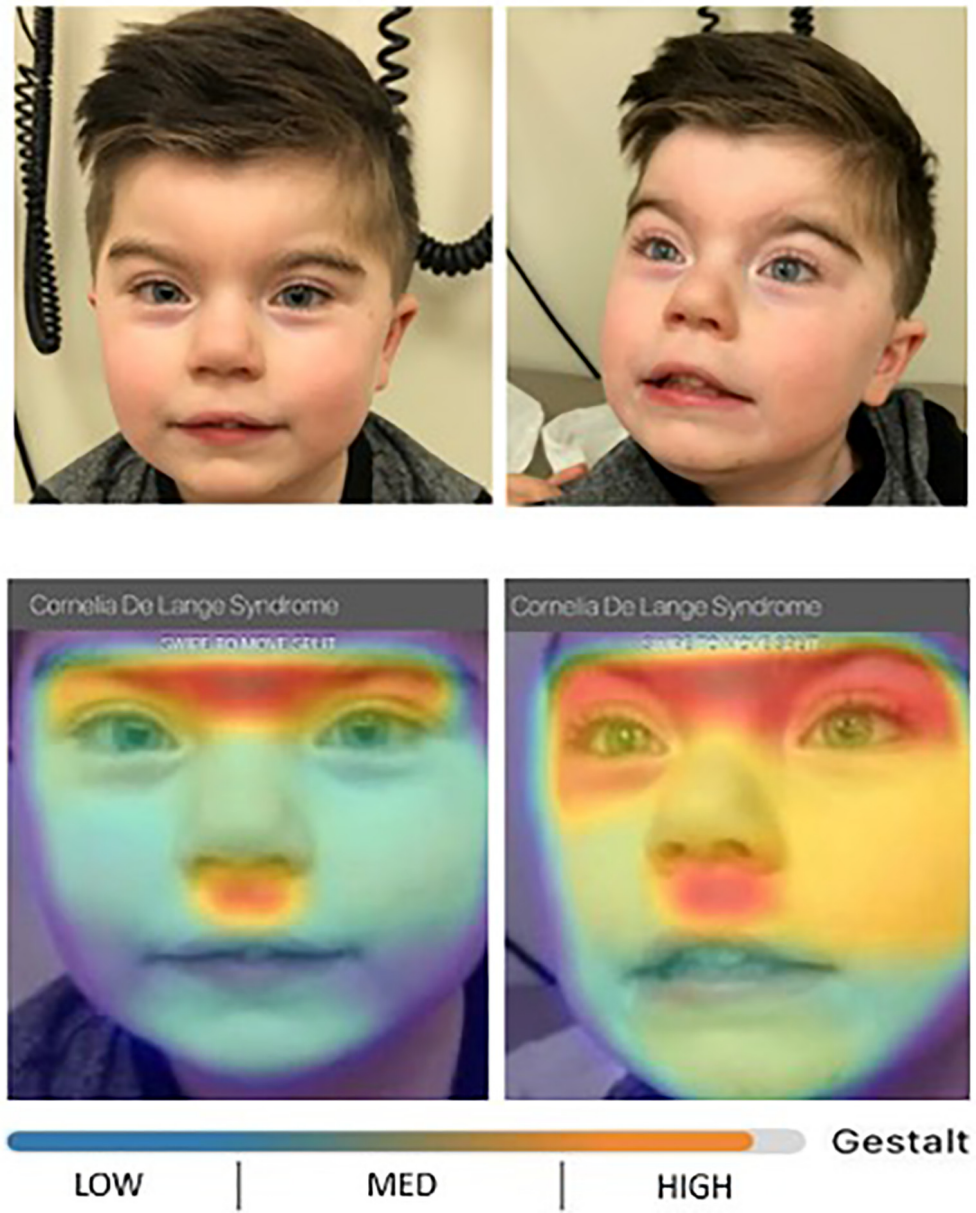
Our patient's facial features and facial heatmap from Face2Gene facial recognition app show high correspondence with Cornelia de Lange gestalt. The distinctive facial features include synophrys, arched eyebrows, long eyelashes, low-set ears, long smooth philtrum, and downturned corners of mouth.

His gestation was complicated by exposure to buprenorphine and pregabalin for an initial 9 wk, alcohol for 10 wk, and tobacco throughout. Normal in utero growth was reported. Patient was born via vaginal delivery at 37 wk gestation to a 22-yr-old primigravida, with a birth weight of 6 pounds. His neonatal course was complicated by meconium aspiration associated with respiratory distress and feeding difficulty due to ankyloglossia. His early developmental milestones were delayed. He sat at 9 mo, pulled to stand at 18 mo, and began walking at around 24 mo. Developmental Assessment of Young Children-2 (DAYC-2) at age 2 indicated significant developmental delays in all domains: gross motor skills (DAYC-2 score 79), fine motor skills (69), receptive and expressive language (64 and 54), social emotional skills (71), and cognition and adaptive skills (70 and 70). He had few words and was not toilet trained by the time of his initial genetics evaluation at 3 yr and 8 mo. Patient had normal head tomography and single-nucleotide polymorphism (SNP) microarray. At the initial genetic evaluation at 3 yr and 8 mo old, his height was 89.10 cm (0.39%ile and −2.66 SDS), weight was 14.10kg (20%ile, −0.84 SDS), and head circumference was 46.1 cm, below the third percentile. His features were reminiscent of the Cornelia de Lange phenotype (see Table 1). Using Face2Gene, a facial digital analysis tool, his gestalt score was a strong match for the CdLS (Fig. 1). Thus, a CdLS next-generation sequencing (NGS)-based gene panel (sequencing and copy-number variant [CNV] analysis), including *NIPBL*, *HDAC8*, *RAD21*, *SMC1A*, and *SMC3*, was ordered on a blood specimen, and the result was negative, with no variants of interest reported. In the interim, the patient was diagnosed with growth hormone deficiency. His initial growth velocity was 5.8 cm/year (10th percentile), but after initiating growth hormone therapy, velocity improved to 8.9 cm/year (90th percentile). The response to growth hormone has not been studied systematically on CdLS patients; however, it is consistent with the experience reported by de Graaf et al. (2017). When the patient returned to the clinic at the age of 5 yr and 1 mo, his height was 100.2 cm, improved to the third percentile (−2.01 SDS). However, microcephaly persisted and his head circumference was 47.5 cm, remaining below the third percentile. In addition, his penis remained relatively small and the length measured at the 10th percentile. Notably, his motor abilities had significantly improved, whereas his speech delay persisted, and he only had about 10 words at 5 yr old and had received an autism spectrum disorder diagnosis. Recent assessment in the context of an individualized education plan, at age 6, reported aggression toward others, requiring constant supervision and maximum teacher cues to complete work at school. He is not toilet trained. He participates in a speech-impaired program and uses assisted technology devices, continuing to qualify for in-school speech and occupational therapy. Given his persistent delays, dysmorphic features, and the negative CdLS sequencing panel, a trio exome sequencing was ordered to look for an alternative diagnosis that mimics CdLS.

**Table 1.** Clinical findings using Human Phenotype Ontology (HPO) term nomenclature (Köhler et al. 2019)

Cornelia de Lange clinical features (HPO terms)	Proband
Microcephaly (HP:0000252)	Yes
Synophrys (HP:0000664)	Yes
Long eyelashes (HP:0000527)	Yes
Low-set ears (HP:0000369)	Yes
Anteverted nares (HP:0000463)	Yes
Long philtrum (HP:0000343) Smooth philtrum (HP:0000319)	Yes
Downturned corners of the mouth (HP:0002714)	Yes
High arched palate (HP:0000218)	No
Widely spaced teeth (HP:0000687)	No
Micrognathia (HP:0000347)	Yes
Short neck (HP:0000470)	No
Growth failure (HP:0001510)	Yes
Developmental delay (HP:0001263)	Yes
Autistic behavior (HP:0000729)	Yes
Abnormality of limbs (HP:0040064)	No
Hirsutism (HP:0001007)	Yes
Gastroesophageal reflux (HP:0002020)	No
Sensorineural hearing impairment (HP:0000407)	No
Ptosis (HP:0000508)	No
Cryptorchidism (HP:0000028)	No
External genital hypoplasia (HP:0003241)	Yes
Novel clinical features	
Hypercholesterolemia (HP:0003124)	Assumed unrelated^a^
Hypertriglyceridemia (HP:0002155)	Assumed unrelated^a^

^a^There have not been any reports of hypertriglyceridemia or hypercholesterolemia in *SMCA1*-caused CdLS so far.

The exome identified two variants of uncertain significance, in *trans*, in a candidate gene, *NSMCE2* (see Table 3). The *NSMCE2* gene encodes a small ubiquitin-like modifier (SUMO) ligase, which plays a role in promoting homologous recombination and DNA damage repair (Potts and Yu 2005). Two patients have been reported by Payne et al. with *NSMCE2* compound heterozygous frameshift mutations; both presented with primordial dwarfism, gonadal failure, and insulin-resistant diabetes, with fatty liver and hypertriglyceridemia. Our patient had some overlapping features with those presented by Payne et al. such as microcephaly and linear growth deficiency. Although liver function and insulin levels on our patient were normal, his fasting lipid profile indicated hypercholesterolemia (total cholesterol 275 mg/dL, reference range <170 mg/dL and LDL cholesterol 201 mg/dL; reference range <110 mg/dL), as well as hypertriglyceridemia (160 mg/dL, reference range <75 mg/dL). But fasting lipid profiles from both parents were normal. We verified with the exome reporting laboratory that there were no reportable variants in known genes causing hyperlipidemia identifiable through exome sequencing. It is uncertain if variants on *NSMCE2* contribute to his hyperlipidemia in any way. Despite some overlapping features, there were significant clinical differences between our patient and those described by Payne et al. as our patient has no signs of insulin resistance or lipodystrophy. Moreover, our patient's facial features were very divergent from those described by Payne et al. who displayed prominent midface, a small jaw, acanthosis nigricans, and skin tags.

At a follow-up clinic visit at 6 yr old, considering the relatively high mosaicism rate of up to 30% reported (Krawczynska et al. 2019) in the clinically diagnosed mutation-negative CdLS patients, a CdLS gene panel (sequencing and CNV analysis), including ANKRD11, HDAC8, KMT2A, NIPBL, RAD21, SMC1A, and SMC3, on buccal swab specimens was ordered and identified a novel hemizygous variant, c.793_795delGAG, p.Glu265del, in *SMC1A* in 60% of sequencing reads (33 of 56 reads), finally provided a definitive molecular diagnosis (Table 2).

**Table 2.** Genomic findings Cornelia de Lange panel (buccal swab)

Gene	Genomic location	HGVS cDNA	HGVS protein	Zygosity	Origin	Variant interpretation	Level of mosaicism	ClinVar accession number
*SMC1A*	Chr X:53,374,148 (GRCh38)	NM_006306.4 c.793_796 delGAG	p.Glu265del	Hemizygous	De novo	Pathogenic variant	60% buccal swab 4% blood 2% blood	SCV001245515

## TECHNICAL ANALYSIS

Cornelia de Lange NGS-based sequencing and a CNV analysis panel (*HDAC8*, *NIPBL*, *RAD21*, *SMC1A*, and *SMC3*) through EGL Genetics Laboratory (EGL) were performed in 2017 with blood as the specimen (please refer to Table 1 in Supplemental Materials for sequencing coverage information). In solution, hybridization of all coding exons within the genes tested was performed on genomic DNA: direct sequencing was done using next-generation short base pair read sequencing. Intronic variants >3 nt from the exon/intron boundary are not reported unless known to be pathogenic.

Trio exome sequencing through GeneDx laboratory was performed in 2018, with blood as the specimen from the proband and saliva as a specimen from both parents (please refer to Tables 2 and 3 in Supplemental Materials for sequencing coverage information). Genomic DNA from the specimen was enriched for coding regions and splice site junctions for most genes on the human genome, sequenced on an Illumina platform, and then filtered and analyzed using their custom analysis tool.

**Table 3.** Genomic findings exome sequencing trio (blood)

Gene	Genomic location	HGVS cDNA	HGVS protein	Zygosity	Parent of origin	Variant interpretation	ClinVar accession number
*NSMCE2*	Chr 8: 8:125,091,778 (GRCh38) Exon 5	NM_173685.2 c.346delT	p.Ser116LeufsX18	Heterozygous	Maternally inherited	Variant of uncertain significance	RCV000412505.1
*NSMCE2*	Chr 8: 125,091,778 (GRCh38) Exon 6	NM_173685.2 c.465T > G	p.Asp155Glu	Heterozygous	Paternally inherited	Variant of uncertain significance	SCV001245059.1

CdLS NGS-based sequencing and a CNV analysis panel (*ANKRD11*, *HDAC8*, *KMT2A*, *NIPBL*, *RAD21*, *SMC1A*, and *SMC3*) through GeneDx laboratory was performed in 2019 with a buccal swab specimen (please refer to Table 4 in Supplemental Materials for sequencing coverage information). The same methodology as trio exome sequencing is used, except sequencing was limited to the genes listed above.

The variant on *SMC1A* was identified by the CdLS sequencing and CNV analysis. The panel was completed with the buccal swab sample in 2019. Upon discussion with GeneDx, which completed exome sequencing in 2018, the variant was detected at 4% mosaicism in blood, which was below the laboratory's 20% threshold for reporting. EGL, which performed Cornelia de Lange panel sequencing in 2017, disclosed that the variant was present in 2% (4 out of 199) of reads in blood specimen. Again, it is below threshold for reporting.

Of note, in 2018, exome sequencing identified compound heterozygous variants of uncertain significance (VUS) on a candidate gene, *NSMCE2* (Table 3).

Variants of the *NSMCE2* gene have been described by Payne et al. (2014) in two female patients with primordial dwarfism, severe insulin resistance, microcephaly, small jaw, fatty liver, hypertriglyceridemia, and ovarian failure.

## VARIANT INTERPRETATION

For the *SMC1A* variant, the criteria considered for classification were Cornelia de Lange–like phenotype, de novo variants not observed in large population cohorts (Lek et al. 2016), in-frame deletion of one amino acid in a nonrepeat region, in silico analysis (including protein predictors and evolutionary conservation) supports a deleterious effect, and has not been previously published as pathogenic or benign.

## SUMMARY

CdLS is a well-known genetic syndrome, recognizable by a distinctive appearance in patients with the classical phenotype, caused by heterozygous pathogenic variants on *NIPBL* in the majority of cases. In cases of milder phenotypes, facial characteristics can be less striking, limb anomalies are often absent, and cognitive impairment is less pronounced. Mild CdLS is more commonly caused by heterozygous pathogenic variants on *SMC3, RAD21*, and hemizygous pathogenic variants in *HDAC8* or *SMC1A.* Somatic mosaicism has been frequently reported in the *NIPBL* gene. These *NIPBL* mosaic cases have generally correlated with a classic appearance and severe manifestations, suggesting that detected mosaicism does not necessarily ameliorate clinical features in this condition, especially for cases caused by variants in *NIPBL* (Castronovo et al. 2010; Gervasini et al. 2013). Somatic mosaicism has been reported less often in other genes that produce a milder CdLS phenotype.

To the best of our knowledge, our case is only the second reported CdLS-like patient with somatic mosaicism on *SMC1A*; this contrasts with high mosaicism on the *NIPBL* gene, which is well known. The scarcity of mosaic cases reported in the *SMC1A* gene could be attributed to its milder and less recognizable clinical features, or variable mosaicism levels in different tissues might be missed on certain types of specimens, as we saw in our patient. In addition, mosaicism levels can vary over time, even on the same tissue. Ansari et al. (2014) reported *SMC1A* mosaicism of 50% and 10%, respectively, in two separate saliva samples of the same individual, producing an atypical phenotype with moderate growth retardation. Furthermore, germline mosaicism has been reported in several cases of the *NIPBL* gene but has also been suggested in a few *SMC1A* cases. They included a family with two affected daughters with a mild CdLS phenotype and parents with negative testing for the familial variant in blood, as well as a family with two affected daughters and a father with qualitative testing (chromatograph), indicative of low-level somatic mosaicism on *SMC1A* and no clinical features of the syndrome (Deardorff et al. 2007; Slavin et al. 2012). All of the above present a challenge for molecular testing strategies and genetic counseling in cases of CdLS-like patients but with an initially negative molecular result.

Here we report a case of somatic mosaicism on *SMC1A* that was detected at 60% in a buccal swab specimen, but detected at only 2% and 4% in two different blood specimens, by retrospectively reanalyzing raw data on previous testing because it is below the reporting threshold. This remarkable difference can be explained by the different origins of DNA in the specimens used. Buccal swabs contain a high proportion of epithelial cells, especially in children, in whom the mean proportion of buccal cells in buccal swabs was 90.3% in a recent study (Theda et al. 2018), whereas leukocytes are the cellular population used in blood samples.

In the current era of clinical genetics, the most cost-effective strategy for diagnosis of syndromes with poorly defined clinical features has become exome sequencing. This approach has the potential to identify new candidate genes in conditions with locus heterogeneity. Our case exemplifies a diagnostic odyssey and illustrates the challenge of somatic mosaicism in clinical practice, as low-level mosaicism can be disregarded by reporting laboratories if below a certain threshold. Our case illustrated that undetected mosaicism is a plausible explanation for unexpected negative molecular results, especially if a blood sample is the only specimen provided for testing. Selection against mutant cells, particularly in lymphocytes, has been suggested as one plausible mechanism and needs to be taken into consideration (Ansari et al. 2014). Fortunately, in CdLS, the computer-assisted facial analysis tool has a high sensitivity for CdLS. A diagnosis of CdLS was within the top five predicted syndromes for 97.9% of cases in a study by LaTorre-Pellicer, and sensitivity was high even for atypical cases and genes other than *NIPBL* (87.5% for *SMC1A*) (LaTorre-Pellicer et al. 2020). Thus, a facial diagnostic tool can assist clinicians to a certain degree in deciding on the best test and specimen to send to increase diagnostic yield.

In conclusion, we suggest selecting different tissues for testing if CdLS is clinically suspected, even after negative results are obtained using a blood specimen, as mosaicism has been frequently reported in this condition.

## ADDITIONAL INFORMATION

### Data Deposition and Access

The SMC1A c.793_796delGAG and NSMCE2 c.465T > G variants were submitted to ClinVar (https://www.ncbi.nlm.nih.gov/clinvar/) and can be found under submission numbers SCV001245515 and SCV001245059.1.

### Ethics Statement

ACMG patient consent for use of medical photography to be used in medical publications, including medical journals, textbooks, and electronic publications, was granted in writing by the parent of the patient.

### Acknowledgments

We thank Cheryl Strauss for her critical review and editing for this manuscript, Erin Torti and the GeneDx ClinVar team for help with the submission of variants to the database, and Audrey Bibb, CGC, for her clinical care and coordination of genetic testing on this patient.

### Author Contributions

H.L. conceptualized the case study, supervised the care of the patient, and critically reviewed and approved the final manuscript, as submitted; A.G.G. directly participated in the patient care, conducted a deep literature search, drafted the initial manuscript, and approved the final manuscript as submitted; and J.M. conducted the literature search and helped with clinical data collection and reviewed and approved the final manuscript, as submitted.

### Competing Interest Statement

The authors have declared no competing interest.

## Supplementary Material

Supplemental Material
