# Supplementary material for: A novel mosaic variant on SMC1A reported in buccal mucosa cells, albeit not in blood, of a patient with Cornelia de Lange–like presentation
Source: Cold Spring Harb Mol Case Stud. 2020 Jun;6(3):a005322. doi: 10.1101/mcs.a005322 (PMC7304356; doi:10.1101/mcs.a005322)
Supplement: Supplemental Material [file supp_6_3_a005322__index.html]

Supplemental Material 

# A novel mosaic variant on *SMC1A* reported in buccal mucosa cells, albeit not in blood, of a patient with Cornelia de Lange–like presentation

## Supplemental Material

- Supplemental\_Material.docx
