# Supplementary material for: A novel mosaic variant on SMC1A reported in buccal mucosa cells, albeit not in blood, of a patient with Cornelia de Lange–like presentation
Source: Cold Spring Harb Mol Case Stud. 2020 Jun;6(3):a005322. doi: 10.1101/mcs.a005322 (PMC7304356; doi:10.1101/mcs.a005322)
Supplement: Supplemental Material [file supp_mcs.a005322_Supplemental_Material.docx]

**Supplemental Information- Coverage Data**

Table S1. Coverage Data Cornelia de Lange: Sequencing Panel (EGL-Eurofins Laboratories)

| **Genes included in Panel** | *NIPBL, HDAC8, RAD21, SMC1A, SMC3* |
| --- | --- |
| **Average Coverage for Panel^1^** | 70x |
| **Low Coverage Threshold (10x)** | No areas of low coverage reported in this test |

^1^ No average coverage data per specific gene is provided by EGL-Eurofins Laboratories

Table S2. Coverage Data Exome Sequencing (XomeDx® Whole Exome Sequencing GeneDx Laboratories)

| **Mean Depth of Coverage^1^** | 118x |
| --- | --- |
| **Quality threshold^2^** | 98.7%* |

^1^ Mean number of sequence reads obtained across the whole exome, specifically the coding exons and splice junctions of protein-coding RefSeq genes that are captured by NextGen sequencing

^2^Percentage of the Exome which is covered by at least 10x

Table S3. Coverage Data for Gene in which variant was reported on Exome Sequencing (XomeDx® GeneDx Laboratories)

| **Gene** | **Chromosomic Location** | **Average Covered at 10x** |
| --- | --- | --- |
| *NSMCE2* | 8q24.1 | 100% |

Table S4. Coverage Data Cornelia de Lange Panel (GeneDx Laboratories)

| **Gene** | **Chromosomic Location** | **Average Covered at 10x** |
| --- | --- | --- |
| *ANKRD11* | 16q24.3 | 99.4% |
| *HDAC8* | Xq13.1 | 99.94% |
| *KMT2A* | 11q23.2 | 99.96% |
| *NIPBL* | 5p13.2 | 99.90% |
| *RAD21* | 8q24.1 | 99.6% |
| *SMC1A* | Xp11.22 | 99.82% |
| *SMC3* | 10q25.2 | 99.98% |
